# SG-APSIC1208: Association between severity of COVID-19 pneumonia and vaccination status in a tertiary-care teaching hospital in Malaysia

**DOI:** 10.1017/ash.2023.8

**Published:** 2023-03-16

**Authors:** Sasheela Sri La Sri Ponnampalavanar, Anjanna Kukreja, Sazali Basri, Sheron Sir Loon Goh, Syifa’ Ahmad Faisol, Reggina Chong Syin Tze, YiShi Ong, Anisa Salleh, SharifahFaridah Syed Omar, Zuhairah Mohd Razali, Adeeba Kamarulzaman

**Affiliations:** University Malaya Medical Centre, Malaysia; University Malaya Medical Centre, Infectious Diseases Unit, Department of Medicine, Kuala Lumpur, Malaysia; University Malaya, Faculty of Medicine, Kuala Lumpur, Malaysia; University Malaya, Faculty of Medicine, Kuala Lumpur, Malaysia; University Malaya, Faculty of Medicine, Kuala Lumpur, Malaysia; University Malaya, Faculty of Medicine, Kuala Lumpur, Malaysia; University Malaya, Faculty of Medicine, Kuala Lumpur, Malaysia; University Malaya, Faculty of Medicine, Kuala Lumpur, Malaysia; University Malaya Medical Centre, Infectious Diseases Unit, Department of Medicine, Kuala Lumpur, Malaysia; University Malaya Medical Centre, Infection Control Department, Kuala Lumpur, Malaysia; University Malaya Medical Centre, Infectious Diseases Unit, Department of Medicine, Kuala Lumpur, Malaysia

## Abstract

**Background and objectives:** Since the introduction of the COVID-19 vaccine through the National COVID-19 Immunization Program in Malaysia in February 2021, the number of cases of severe COVID-19 and mortality have progressively decreased. We explored the association between vaccination status, type of vaccine, and the highest COVID-19 clinical category. **Methods:** Patients were recruited via the electronic medical record (EMR) at University Malaya Medical Centre (UMMC) from July 2021 onward. Included patients were aged ≥18 years old with positive SARS-CoV-2 RT-PCR results from respiratory samples (naso-oropharyngeal swab, saliva, or sputum). Patient demographic data, COVID-19 clinical category, vaccination status, and type of vaccine received were recorded. **Results:** In total, 1,391 positive SARS-CoV-2 PCR results were reviewed; 1,188 patients (85%) with complete data were analyzed. These patients’ median age was 50 years. The proportions of patients COVID-19 clinical categories were as follows: category 1 (4.04%), category 2 (28.37%), category 3 (10.7%), category 4 (30.6%), and category 5 (2.6%). The mortality rate was 21.5%. As of July 2021, only 16.8% of patients were fully vaccinated, 30.3% were vaccinated, 31.5% unvaccinated, and 21.5% had unknown vaccination status. In total 364 patients with category 4 COVID-19 (4.4%; *P* < .001) were fully vaccinated and no patients who were fully vaccinated had category 5 COVID-19 (*P* = .011). Furthermore, 40.8% of patients who died had unknown vaccination status (*P* < .01); 28.1% of patients who died were unvaccinated (*P* = .015); 25.3% of patients who died were partially vaccinated (*P* = .036); and 0.4% of patients who died were fully vaccinated (*P* < .001). For category 4 and 5 illness and death, there were no significant differences between the type of vaccine received (Pfizer-BioNTechR, Astra ZenecaR and Coronavac/SinovacR) and severe COVID-19. **Conclusions:** The completion of 2 doses of government-approved COVID-19 vaccination is paramount in preventing severe COVID-19 disease and death. Rapid rollout and equitable distribution of vaccination should be initiated. Vaccine hesitancy should be promptly addressed to ensure vaccination uptake.

